# Work Status and Return to the Workforce after Coronary Artery Bypass Grafting and/or Heart Valve Surgery: A One-Year-Follow Up Study

**DOI:** 10.1155/2014/631842

**Published:** 2014-06-15

**Authors:** Kirsten Fonager, Søren Lundbye-Christensen, Jan Jesper Andreasen, Mikkel Futtrup, Anette Luther Christensen, Khalil Ahmad, Martin Agge Nørgaard

**Affiliations:** ^1^Department of Social Medicine, Center for Cardiovascular Research, Aalborg University Hospital, 9100 Aalborg, Denmark; ^2^Department of Health Science and Technology, Faculty of Medicine, Aalborg University, 9220 Aalborg, Denmark; ^3^Department of Cardiothoracic Surgery, Center for Cardiovascular Research, Aalborg University Hospital, 9100 Aalborg, Denmark; ^4^Department of Clinical Medicine, Aalborg University, 9100 Aalborg, Denmark

## Abstract

*Background*. Several characteristics appear to be important for estimating the likelihood of reentering the workforce after surgery. The aim of the present study was to describe work status in a two-year time period around the time of cardiac surgery and estimate the probability of returning to the workforce.* Methods*. We included 681 patients undergoing coronary artery bypass grafting and/or heart valve procedures from 2003 to 2007 in the North Denmark Region. We linked hospital data to data in the DREAM database which holds information of everyone receiving social benefits.* Results*. At the time of surgery 17.3% were allocated disability pension and 2.3% were allocated a permanent part-time benefit. Being unemployed one year before surgery reduced the likelihood of return to the workforce (*RR* = 0.74 (0.60–0.92)) whereas unemployment at the time of surgery had no impact on return to the workforce (*RR* = 0.96 (0.78–1.18)). Sickness absence before surgery reduced the likelihood of return to the workforce.* Conclusion*. This study found the work status before surgery to be associated with the likelihood of return to the workforce within one year after surgery. Before surgery one-fifth of the population either was allocated disability pension or received a permanent part-time benefit.

## 1. Background

In the early era of cardiac surgery, the main focus was on immediate postoperative survival. The EuroScore (the European System for Cardiac Operative Risk Evaluation) is today probably the most widely implemented scoring system for estimating mortality up to 30 days after surgery [1]; however, with improving results regarding survival, more attention should now be paid to the postoperative quality of life, including the patients' ability to return to the workforce. The ability to return to the workforce must be regarded as a very important part of the postoperative outcome since many patients are still part of the workforce when they undergo cardiac surgery.

Several clinical and sociodemographic factors have been associated with return to the workforce for patients undergoing cardiac surgery, for example, occupation, relief of symptoms, age, and education [2–9]. As expected, the rate of patients returning to work has been found to be lower in patients with comorbidity than in patients with no comorbidity [10] and certain psychological variables have been shown to be important as well [11]. Participation in cardiac rehabilitation programs have been found to be associated with an increasing number of patients returning to work compared with patients not joining such programs [12]. Finally, preoperative work status might also have an impact on the postoperative likelihood of return to work [2, 4–6, 8].

The aim of the present study was to describe work status in a two-year time period around the time of surgery and estimate the probability of returning to the workforce, in Denmark, depending on work status before surgery.

## 2. Methods

The Danish National Health Service provides tax-funded healthcare and social welfare for all citizens. By use of the unique civil registration number (CPR number) assigned to all Danish citizens, unambiguous linkage between various registers and databases can be performed. This cohort study was conducted within the population of the North Denmark Region (population: 0.6 million) and the study was approved by the Danish Data Protection Agency.

All patients undergoing cardiac surgery in the North Denmark Region are included in the Western Denmark Heart Registry [13]. This registry keeps records of all procedures and operations performed on patients admitted for adult cardiac surgery in the western part of Denmark. Data in this database are registered by the departments at the time of hospitalisation (preoperative data) and by the surgeons, anesthesiologists, and perfusionists (operative data).

The material for this study was consecutive patients receiving first-time coronary artery bypass grafting (CABG) and/or heart valve-procedures. Patients receiving re-do surgery or other concomitant procedures were not included in the study. The patients were operated on during the period from January 1, 2003, to December 31, 2007.

From the Western Denmark Heart Registry we extracted information on all patients aged 62 years or younger at the time of surgery. This age was chosen so the patients had a potential of at least two years of workforce association, after surgery, before ordinary retirement (the retirement age was changed between 65 and 67 years during the observation period). From the database we included information regarding type of operation and the logistic EuroScore I. This score is based on patient-related factors (i.e., neurologic dysfunctional disease, serum creatinine, etc.), heart-related factors (i.e., unstable angina, left ventricular dysfunction, etc.), and operative factors (i.e., other surgeries than isolated CABG, postinfarct septal rupture, etc.) [1].

In Denmark, social security benefits and social services are financed by taxation and all citizens in need are entitled to receive social security benefits and social services—regardless of factors such as their affiliation to the labour market. Short-term sickness benefit (in the study period first two weeks of sick leave) is paid by the employer, and thereafter by the municipality sometimes with a supplement from the employer [13]. Both Self-employed and employees are covered.

Using the unique civil registration number of the patients data were linked to the DREAM database where information on all social benefits is registered on a weekly basis. The DREAM database contains pooled data from all relevant Danish ministries, all Danish municipalities and the national bureau of statistics (Statistics Denmark) since July 1991. This database has been found suitable for public health research [14, 15]. The DREAM database has more than 100 different codes which cover benefits paid to a citizen at any given week. Short-term sick-listing (in the study period less than two weeks) is usually not recorded for working citizens. For the present study we used codes for unemployment (including both benefits for patients with and without private unemployment insurance), sick leave, part-time benefit (flexjob, i.e., job created for persons with a permanent limited working capacity), and disability pension. Patients registered with none of these codes were classified as working. Patients registered with codes, for example, national education grants and early retirement benefits were considered self-supporting and classified in the working group.

Work status was defined by use of DREAM data. We categorized the patients into five groups before and after surgery as follows.

1. Working.
2. Unemployed.
3. On sick leave.
4. Receiving part-time benefit.
5. Receiving disability pension.

For each patient we extracted the information from the DREAM database from one year before surgery till one year after.

### 2.1. Statistical Analysis

The primary endpoint was return to the workforce (defined as being capable to work, that is, working or unemployed 6 months or 12 months after surgery). The objective was to describe the importance of work status (measured by DREAM data) before surgery. Two associations were investigated. Firstly we studied the importance of work status from nine to twelve months before surgery (the main type of benefit in the three months' time period) on return to the workforce. Secondly we studied the influence of work status the week before surgery on return to the workforce.

The relative risks were presented unadjusted and adjusted for EuroScore (included as a continuous variable), type of operation (CABG, valve operation, or both), age (three groups), and gender using a modified Poisson regression model [16].

Statistical tests were two-tailed, and *P* < 0.05 was considered significant. Statistical analyses were performed using Stata version 11.2 (StataCorp. 2009. Stata: Release 11. Statistical Software. College Station, TX: Stata Corp LP, USA).

## 3. Results

A total of 681 patients operated on during the study period fulfilled the inclusion criteria.  Table 1 shows the characteristics of the study population. Most of the patients were men and the age varied between 19 and 62 years. Most of the study population underwent CABG either alone or in combination with valve surgery. One year before surgery 30% of the women and 17% of the men had been allocated either disability pension or permanent part-time benefits and the week before surgery this percentages was 33% and 18%, respectively. In total, 17.3% were allocated disability pension and 2.3% were allocated a permanent part-time benefit before surgery. Overall, less than 5% were on sick leave one year before surgery and one week before surgery this figure increased to 45%.

**Figure 1:**
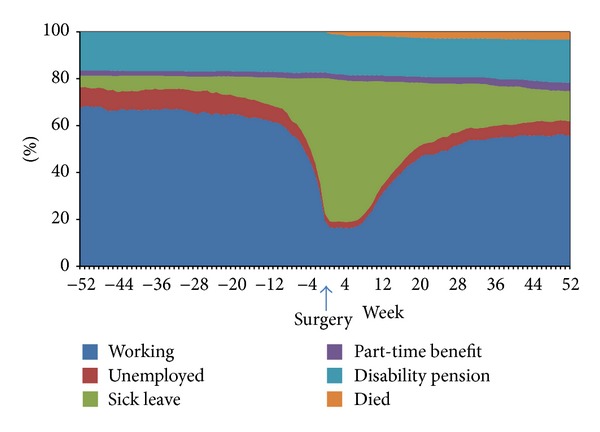
Work status for patients undergoing coronary artery bypass surgery and/or heart valve surgery in the North Denmark Region from one year before surgery to one year after.

The percentage of patients on sick leave increased from especially two months before surgery, and at the first weeks after surgery more than 60% of the study population was on sick leave (Figure 1). It is noteworthy that about 16% of the study population did not receive any social benefits around the time of surgery. During the one-year follow-up 22 patients died; nine of them were allocated disability pension or part-time benefits before surgery.

Overall 55% were working or unemployed 6 months after surgery which increased to 62% 12 months after surgery (Table 2). Patients receiving disability pension or part-time benefits before surgery remained on these benefits during the study period.

Being unemployed one year before surgery reduced the likelihood of returning to the workforce (*RR* = 0.74 (0.60–0.92)) whereas unemployment at the time of surgery had no impact in the adjusted model (*RR* = 0.96 (0.78–1.18)) (Table 3). Being on sick leave both one year before and at the time of surgery reduced the likelihood of returning to the workforce but was most pronounced for patients on sick leave one year before surgery (*RR* = 0.52 (0.34–0.78)). 13 out of the 22 patients who died during follow-up did not receive any permanent benefits at the time of surgery. Excluding those who died during the follow-up period from the analyses did not change the relative risks.

## 4. Discussion 

The study showed that one-fifth of the study-group below the age of 63 was allocated either disability pension (17.3%) or a permanent part-time benefit (2.3%) before they underwent CABG and/or valve surgery. Patients being unemployed or on sick leave one year before surgery had a reduced likelihood of returning to the workforce after surgery, whereas unemployment at the time of surgery had no impact. Being on sick leave at the time of surgery had only a minor impact on returning to the workforce one year after the surgery.

How often the patients return to the workforce after cardiac surgery varies from less than one third to no difference in employment rate one year after surgery compared to before [3–8, 10–12, 17, 18] which might be explained by differences in study populations, time periods, and the socioeconomic support system of the countries. The proportion of patients returning to the workforce in our study was especially high if the patients were working and the lowest for patients at sick leave one year before surgery. The importance of work status before cardiac surgery for returning to the workforce postoperatively has previously been demonstrated [3–6, 8]. One obvious explanation lies in differences in health status between those who work and those who do not, especially looking at patients on sick leave one year before surgery. By including the EuroScore we tried to adjust for the differences in health status among the patients prior to surgery. However, EuroScore is an implemented scoring system for estimating postoperative mortality [1], and the EuroScore alone may not have been able to fully adjust for all comorbidities, for example, diabetes, adiposity, musculoskeletal disorders, and psychiatric diseases which might have an impact on the patients' ability to reenter the workforce postoperatively. Therefore, there is a risk of residual confounding with respect to comorbidities.

Not only being on sick leave but also being unemployed the year before surgery had a significant negative impact on the chance of returning to the workforce one year after surgery and might indicate that health status alone is not the only factor critical to the patients chance of returning to the workforce. Unemployment has earlier been associated with an increased risk of all-cause mortality but the mechanism is not fully understood [19]. However, a recent study indicated that people with impaired health are forced out of the labor market in times of increasing unemployment rather than pointing towards a negative effect of unemployment on health [20].

The strength of this registry-based study is the uniform data collection at baseline and the complete follow-up which minimizes the risk of selection bias. We had no information regarding the type of work or education which might have had an impact on the patients' chance of returning to the workforce [21]. Furthermore we only had information about the type of benefits the patients received, but not the reason (i.e., the reason for being on sick leave).

The patients were included from 2003 to 2007 and followed the first year after surgery. Since then the number of available jobs in general has been reduced due to the financial crisis. However, focusing on return to the workforce instead of return to work probably made the estimates more robust for the one-year follow-up. Furthermore, the influence of the financial crisis on changes in the social security system would more likely have an impact looking at longer follow-up periods.

It is surprising that 16% of the study population did not receive any social benefit at the time of surgery. This may indicate short-term sick leave for these patients, since only sick leave more than two weeks is registered in the DREAM database. Some of the patients not receiving benefit may reflect that they were economically funded by their spouses. However, this figure is probably low.

In the postoperative period patients may experience fatigue, anxiety, depression, and cognitive dysfunction [22, 23] and these conditions may influence the ability of the patients to return to the workforce. An observational study from Israel indicated more frequent return to the workforce among participants in a cardiac rehabilitation programmes, in a population with a participation rate at 7% [12]. In Denmark complete participation in cardiac rehabilitation has been found even lower for patients with ischaemic heart disease [24]. More knowledge regarding the impact of cardiac rehabilitation on the possibility to return to the workforce is needed among patients who undergo cardiac surgery. Furthermore, the impact of culture and socioeconomic factors present in different countries should be analysed in future studies.

In conclusion, this study found work status before surgery to be important for the likelihood of returning to the workforce after CABG and/or valve surgery. Furthermore, at the time of surgery 17% of patients below the age of 63 were allocated disability pension.

## Figures and Tables

**Table 1:** Characteristics of patients undergoing coronary artery bypass surgery and/or heart valve surgery in the North Denmark Region, from 2003 to 2007; numbers and percentages.

		Men	Women
		*N* = 581	N = 100
		*n* (%)	*n* (%)
Age	Median (range)	*56 (19–62) *	*55.5 (22–62) *

Type of operation	CABG∗	444 (77)	57 (57)
	Heart valve surgery	107 (19)	32 (32)
	Both	30 (5)	11 (11)

EuroScore∗∗	Median (range)	1.5 (0.9–52.3)	2.4 (1.2–61.9)

Work status 9–12 months before surgery	Working	409 (70)	51 (51)
	Unemployed	46 (8)	11 (11)
	On sick leave	28 (5)	8 (8)
	Receiving part-time permanent benefit∗∗∗	11 (2)	3 (3)
	Disability pension	87 (15)	27 (27)

Work status the week before surgery	Working	185 (32)	28 (28)
	Unemployed	23 (4)	5 (5)
	On sick leave	272 (47)	34 (34)
	Receiving part-time permanent benefit∗∗∗	12 (2)	4 (4)
	Disability pension	89 (15)	29 (29)

*CABG: coronary artery bypass grafting. ∗∗A scoring system for estimating the 30-day mortality based on patient-related factors, cardiac-related factors, and operation related factors. ∗∗∗A benefit awarded if the applicant has a permanently reduced ability to work.

**Table 2:** Return to the workforce 6 and 12 months after surgery for patients undergoing CABG and/or heart valve surgery in the North Denmark Region from 2003 to 2007.

		Return to the workforce (working or unemployed)∗			
		6 months after surgery		12 months after surgery	
		Yes	No	Yes	No
		*N* (%)	*N* (%)	*N* (%)	*N*(%)
Overall		376 (55)	305 (45)	420 (62)	261 (38)

Work status 9–12 months before surgery	Working	331 (72)	129 (28)	373 (81)	87 (19)
	Unemployed	33 (58)	24 (42)	33 (58)	24 (42)
	On sick leave	12 (33)	24 (67)	14 (39)	22 (61)
	Receiving part-time permanent benefit	0 (0)	14 (100)	0 (0)	14 (100)
	Disability pension	0 (0)	114 (100)	0 (0)	114 (100)

Work status the week before surgery	Working	177 (83)	36 (17)	183 (86)	30 (14)
	Unemployed	23 (82)	5 (18)	21 (75)	7 (25)
	On sick leave	176 (58)	130 (42)	216 (71)	90 (29)
	Receiving part-time permanent benefit	0 (0)	16 (100)	0 (0)	16 (100)
	Disability pension	0 (0)	118 (100)	0 (0)	118 (100)

*22 patients died during the one year follow-up (9 working, 3 unemployed, 1 on sick leave, and 9 receiving part-time benefit or disability pension one year before surgery), included in the No category.

**Table 3:** The chance of having returned to the workforce 12 months after coronary artery bypass surgery and/or heart valve surgery, by work status before surgery.

		Return to the workforce (working or unemployed)		
		*N* (%)	*RR* (95% CI)	RR* (95% CI)
Work status 9–12 months before surgery	Working	373 (81)	1	1
	Unemployed	33 (58)	0.71 (0.57–0.89)	0.74 (0.60–0.92)
	On sick leave	14 (39)	0.48 (0.32–0.72)	0.52 (0.34–0.78)

Work status one week before surgery	Working	183 (86)	1	1
	Unemployed	21 (75)	0.87 (0.70–1.09)	0.96 (0.78–1.18)
	On sick leave	216 (71)	0.82 (0.75–0.90)	0.85 (0.78–0.94)

*Adjusted for age, gender, type of operation, and EuroScore.
